# Congenital ocular and its adnexal anomalies among Indian pediatric age groups

**DOI:** 10.6026/973206300200323

**Published:** 2024-04-30

**Authors:** Preethi Chava, Chaitra MC, Raveena J, Amulya Padmini

**Affiliations:** 1 Department of Ophthalmology, Sri Devaraj Urs Medical College, Tamaka, Kolar, India

**Keywords:** Congenital anomaly, nasolacrimal duct blockade, maternal risk factors, paediatric age, consanguinity

## Abstract

An analysis of the congenital etiologies of ocular morbidity in children of age 0-12 years is of interest. Hence, this study was conducted over a period of 2 years from Jan 2021- Dec 2023 at RL Jalappa Hospital and Research center that is attached to Sri Devaraj Urs Medical College, Tamaka, Kolar, Karnataka, India. Out of 56 patients, 57% were male and 43% were female children. 31 (55%) of mothers belonged to age group between 20-30 years and 24 (43%) between 31-40 years and 1(2%) between 41-50 years. Out of 56 patients, 14 (25%) of them had positive family history. 34 (61%) of them had consanguious marriage. 14 parents (41%) out of 34 are married to second degree consanguinity (brother/sister/grandparent/grandchild) and 20 (59%) belonged to third degree consanguinity (aunt/uncle/niece/nephew/great-grandparent/great-grandchild). Bilateral involvement was seen in 31 (55%). Nasolacrimal duct anomalies were found to be the most common (32%) followed by congenital esotropia (14%). Education, awareness, counseling about risks of consanguinity and other risk factors such as maternal age, infections, medications during pregnancy, vaccination must be a routine practice in healthcare set up. This can significantly reduce morbidity and prevent blindness.

## Background:

Congenital abnormalities are one of the important causes of ill health and death in children worldwide. These anomalies encompass a spectrum of abnormalities or malformations affecting the eye or its surrounding structures present at birth. They vary in severity, ranging from minor issues to severe conditions that can lead to considerable visual impairment or blindness if untreated. Globally, major structural childhood blindness conditions such as ano-phthalmos, micro-phthalmos, and coloboma account for 16.7% of total childhood blindness cases. [1] The loss of vision can impact various aspects of life including quality of life, independence, and mobility. It has been associated with increased risks of falls, injury, and deterioration across mental health, cognition, social functioning, employment, and educational achievement. [2] Illiteracy, poor socioeconomic conditions, and lack of basic health facilities all contribute to the already large issue of visual impairment in children of third world countries. All of these factors can lead to irreversible blindness despite preventable conditions in many cases [3]. Universal newborn eye screening is evidently effective in detecting numerous abnormalities during the initial ophthalmic examination, which would otherwise remain undetected without such screening. [4] A comprehensive understanding of the developmental pathogenesis of congenital ocular anomalies is essential for accurate diagnosis and timely intervention. Adequate history-taking during patient evaluation enables the adoption of preventive measures, particularly considering the significant impact of maternal infections and systemic involvement in this context. The fundamental morphogenetic stages of eye development conclude by the end of the second month of gestation, with full maturation occurring post-natally. Any disruption during this developmental process can result in various congenital ocular anomalies. Significant factors contributing to these anomalies include intrauterine infections, medication exposure, consanguineous marriages, and maternal metabolic disorders such as folic acid deficiency, diabetes, cretinism, and alcoholism. Congenital ocular anomalies stand as a significant contributor to childhood blindness, with major structural anomalies like ano-phthalmos, micro-phthalmos, and coloboma accounting for 16.7% of total childhood blindness cases worldwide. [5] Hence, it is essential to have a basic epidemiological data on these anomalies. Therefore, it is of interest to analyse the congenital etiologies of ocular morbidity in children of age 0-12 years.

## Methodology:

## Study design:

Prospective observational study

## Time period:

2 years from Jan 2021-Dec 2023

## Sample size:

The sample size (56) is estimated based on one of the most common ocular pathology that is refractive error in a study by Singh *et al.* [6]. The estimated sample size is 56 with 95 % confidence using 'n' Master 2.0 software.

## Study population:

All cases of age group 0-12 years attending the OPD and camps with ocular symptoms

## Inclusion criteria:

Age: 0-12 years

## Exclusion criteria:

[1] Children whose parents refuse to give consent

[2] Children who are systemically unstable and cannot be examine

After obtaining an assent from the child and parental permission from the child's parents, child will be subjected to screening which includes name, age, sex, address, detailed antepartum, intrapartum and post-partum history such as history of consanguinity, history of infections and history of radiation, consumption of drugs in the perinatal period ,history of method of delivery, full term or premature ,history of any infections to the child after delivery, and family history of ocular abnormalities will be considered. APGAR score will be noted in cases where necessary.

A complete general physical examination will be done to rule out other congenital abnormalities of the heart, gastro intestinal tract, spine, upper and lower limbs, face and cranium. Red reflex testing will be done for all the new born babies to rules out causes of leukocoria such as retinoblastoma, persistent primary hyperplastic vitreous, retinal detachment etc. Rough estimation of visual acuity will be done in new born babies by torch light examination, children of age 2-5 years will undergo illiterate E cut out test, and children above age 5 will undergo visual acuity assessment by snellen's visual acuity chart. The periorbital area, orbital rim, lids and adnexa, anterior segment evaluation will be performed by diffuse light examination using a stand flash light or slit lamp examination whenever necessary. Posterior segment evaluation will be done using a direct ophthalmoscope or under slit lamp bio-microscopy using a +90D lens or a +115 D lens or by indirect ophthalmo-scopic examination using a +20 D lens. B-scan will be performed in cases with severe corneal edema or any media opacities obscuring the fundus evaluation. Optical coherence tomography will be performed in cases necessary.

Any case requiring further investigations to rule out buphthalmos and congenital nasolacrimal duct obstruction such as gonio-scopy, indentation tonometry and probing respectively, will be taken under general anaesthesia. A case of congenital ptosis will be evaluated thoroughly which includes assessment of bell's phenomenon, levator function and extra ocular movements. Cover, uncover and alternate cover tests will be performed in a case of squint. General investigations like complete hemogram, electrocardiogram, X ray of the chest and spine, echocardiography, ultra-sonogram of the abdomen and pelvis will be performed to rule out other congenital abnormalities.

## Statistical analysis:

Quantitative measurements were represented in mean and standard deviation, categorical variables in percentage. The 'chi-square' test was used to measure difference in proportion. 'p' value of <0.05 was considered as statistically significant.

## Results:

Out of 56 patients, 32 (57%) were male and 24 (43%) were female (Table 1). Among the risk factors 31 (55%) of mothers belonged to age group between 20-30 years, 24 (43%) between 31-40 years & 1(2%). 14 (25%) of them had positive family history. 34(61%) of them had consanguious marriage and the degree of consanguinity was: 10 (30%) belonged to first degree, 11 (32%) belonged to second degree & 13 (38%) to third degree (Table 2). Bilateral involvement was seen in 31 (55%). Nasolacrimal duct anomalies were found to be the most common (32%) followed by Congenital esotropia (14%), congenital cataract (11%), coloboma of uveal tract 7%), persistent pupillary membrane (5%), micro-phthalmos (2.0%), congenital glaucoma (2%), congenital ptosis (2%), heterochromia iridis (2%), limbal dermoid (2), dermoid cyst (4%), Optic nerve hypoplasia( 4%)% & telecanthus/epicanthus (4%) of total eyes (Table 3).

## Discussion:

The proportion of males affected was significantly higher than females (Z = 3.95, P < 0.001). This is similar to Dash *et al.* [7]. Bilateral (55.35%) involvement more than unilateral cases (44.65%). Most common causes for bilateral anomalies are congenital NLD obstruction and uveal tract coloboma. Further, the common cause for unilateral anomalies is micro-phthalmous followed by congenital ptosis. LO-COA *et al.* [8] shows unilateral visual impairment micro-phthalmos (5.5%). This is similar to our data with unilateral involvement (2%). Panda *et al.* [9] study found that most common disorder was CNLDO. Our study has also shown congenital NLD obstruction was the most common cause (32%). Various studies report the prevalence of congenital NLDO varies from 5 % to 20 % in early childhood. Bilateral is more common in NLD obstruction [8]. Tupe *et al.* [10] observed that in 18 cases (36%) parents gave a history of consanguinity, of these 16% had 1st degree of consanguinity, 78% had 2nd degree consanguinity and only 6% subject's 3rd degree consanguineous relations in marriage. Our data also showed history of consanguinity, 30% belonged to 1st degree, 32% belonged to 2nd degree and 38% belonged to 3rd degree.

In syed *et al.* [11], 11 % found to have some form of coloboma of uveal tract and 7 % had congenital glaucoma. Our study shows 7% of them had coloboma of uveal tract and 2% has congenital glaucoma. However, the incidence of coloboma is quite high (20%) in Behera *et al.* [5]. A study done in 2019 showed 29.3% of cases associated with congenital cataract and in 2024 with advanced medical diagnosis, parents and family members generally detecting leukocoria and initiating treatment early, in this study it showed only 11% of congenital cataract. Congenital cataract remains the most common treatable cause of visual disability in infancy and childhood. Monudi *et al.* [12] showed congenital ptosis was the commonest malformation seen and accounted for 15.4%, with 2/3 of the cases being unilateral but this was contrary to our study which showed only 1% of total cases.

Sarosh *et al.* found esotropia was highly prevalent in approximately 60%. In our study 14% of patients presented with congenital esotropia. Congenital esotropia is usually missed but early correction can effectively reduce morbidity [13]. Persistent pupillary membrane results from the persistence of a portion of the anterior vascular sheath of the lens, which can be attached to either the anterior surface of the iris or the posterior surface of the cornea, mostly these remnants are small, doesn't require treatment and these disappear or shrink by 1st years of life and our data showed 5% incidence. Tupe *et al.* [10] showed 5 cases micro-phthalmos were detected, which make prevalence of 0.5 per thousand populations and our data showed 1 case (5%).

This study is one of the few from India to report on congenital eye anomalies in children. It is an observational study with a small sample size and lack of genetic studies, thereby hindering the generalizability of its findings. In conclusion, congenital ocular anomalies, notably congenital nasolacrimal duct obstruction (CNLDO) and congenital cataract, are prevalent causes of ocular morbidity among children and treatable. Early diagnosis, screening, timely referral from community levels and appropriate intervention are essential to mitigate permanent sequelae and morbidity associated with congenital ocular anomalies.

## Conclusion:

Data shows that congenital NLD obstruction was the common cause for bilateral anomaly and micro-phthalmous as the common cause for unilateral anomaly. Hence, education, awareness and counseling about risks of consanguinity and other risk factors such as maternal age, infections and medications during pregnancy and vaccination must be a routine practice in healthcare set up is necessary. This can significantly reduce morbidity and prevent blindness. Proper knowledge of the developmental pathogenesis of congenital ocular anomalies is highly important for correct diagnosis and early intervention. Preventive measures can be adopted if history is taken properly during evaluation of the patients because maternal infection and systemic involvement have a great impact in this context.

## Figures and Tables

**Table 1:** Gender data

**Gender**	**Frequency (n= )**	**Percentage**
Male	32	57%
Female	24	43%

**Table 2:** Risk factors

**Risk factor**	**Frequency (n=)**	**Percentage**
Maternal Age 20-30 years	31	55%
31-40 years	24	43%
41-50 years	1	2%
**Family history**		
Yes	14	25%
No	42	75%
Consanguinity	34	61%
**Degree of consanguinity**		
2nd	14	41%
3rd	20	59%

**Table 3:** Type of Ocular anomaly

**Congenital ocular anomaly**	**Both eyes**	**Right Eye**	**Left Eye**	**Total**	**Percentage (n= 56 )**
Congenital cataract	2	2	2	6	11
Congenital NLD block	12	2	4	18	32
Coloboma Iris	4	-	-	4	7
Microphthalmos	-	1	-	1	2
Microcornea	2	-	-	2	4
Congenital Glaucoma	1	-	-	1	2
PPM	1	2	-	3	5
Limbal dermoid	-	-	1	1	2
Dermoid cyst	2	-	-	2	4
Optic nerve hypoplasia	2	-	-	2	4
Heterochromia iridis	1	-	-	1	2
Choroidal coloboma	4	-	-	4	7
Congenital Ptosis	-	1	-	1	2
Congenital esotropia				8	14
Epicanthus / Telecanthus				2	4
